# Shaping Thermal Transport and Temperature Distribution via Anisotropic Carbon Fiber Reinforced Composites

**DOI:** 10.1021/acsomega.4c06558

**Published:** 2024-09-04

**Authors:** Flora Lebeda, Martin Demleitner, Annalena Pongratz, Holger Ruckdäschel, Markus Retsch

**Affiliations:** † Department of Chemistry, Physical Chemistry I, University of Bayreuth, Universitätsstraße 30, 95447 Bayreuth, Germany; ‡ Bavarian Center for Battery Technology (BayBatt), Weiherstraße 26, 95448 Bayreuth, Germany; § Department of Polymer Engineering, University of Bayreuth, Universitätsstraße 30, 95447 Bayreuth, Germany; ∥ Bavarian Polymer Institute and Bayreuth Institute of Macromolecular Research, Universitätsstraße 30, 95447 Bayreuth, Germany; ⊥ Bavarian Polymer Institute, Bayreuth Center for Colloids and Interfaces, Universitätsstraße 30, 95447 Bayreuth, Germany

## Abstract

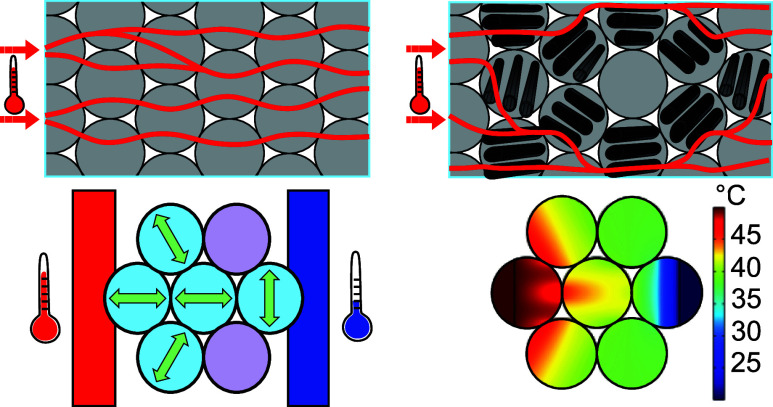

With the ongoing electrification of vehicles, thermal management is on everyone’s lips. To prevent overheating in electronic systems, new design strategies for thermal dissipation are needed. Thermally anisotropic materials enable targeted directional heat transport due to their anisotropic thermal conduction. Laminates made of unidirectionally aligned carbon fibers in a polymer matrix can be tailored regarding their in-plane anisotropy. Exposing the laminates to a temperature gradient reveals that the thermal transport is determined by their anisotropic properties. The corresponding heat flow can be visualized by IR thermography. The combination of anisotropic laminate discs into composite materials, similar to building with toy bricks, enables precise control of heat transport in the macroscopic composite materials. Thus, we achieve control of heat flow at the level of the individual components. In addition, we show that the orientation of anisotropy relative to the temperature gradient is crucial to guide the heat flow selectively. We found that the ratio of thermal anisotropy, the amount and arrangement of anisotropic components, and their positioning in the composite strongly influence heat transport. By combining all these factors, we are able to locally control the heat flow in composites by creating materials to either dissipate heat or block heat transport. The proposed concept can be extended to different shapes of building blocks in two or three dimensions.

## Introduction

Temperature as a measure of the mean kinetic energy of particles in a system is an essential characteristic in many fields.^1,2^ It fundamentally determines the physical and chemical properties of matter. Innovative heating and cooling techniques have been developed to manipulate systems to achieve specific temperature conditions. In industry, the supply or dissipation of heat is crucial for achieving or maintaining optimal operating temperatures.^3,4^ Above all, excessive temperature loads and overheating can cause serious damage to electronic components.^5^ One of the main sources of failure in electronic systems is thermal excitation, i.e., the direct application of heat.^6−8^ Overheating issues are triggered by the imperative to miniaturize power elements, ever-stronger enclosures of electronic devices, and constrained space for heat dissipation by convection.^9^ Consequently, increasing emphasis is placed on the thermal design when developing components. In general terms, heat management is achieved via highly insulating or highly conducting composites.^10−14^ Both—admittedly contrary—goals are often achieved by structured or composite materials, e.g., foams for thermal insulators, and percolating networks for heat dissipation.^11,15,16^ Typically, the challenges for cooling, which requires highly conducting materials, are considerably larger than for heating. In this context, the emergence of thermally anisotropic materials presents opportunities for more precise thermal management.^17−19^ Here, high and low thermal conductivity are combined in one material but are distinguished by their directionality.

Direction-dependent properties are ubiquitous in our environment, with nature providing a surprising variety of anisotropic materials, ranging from microstructure (anisotropic crystal planes,^20^ fibers^21^) to bones,^22^ dental tissues,^22^ wood, and rocks^23^ in the macroscopic world. Human-made examples include composites,^24^ paper and textiles.^25^ Many newly developed materials also exhibit anisotropic properties.^24,26^ Superior anisotropy is gained by the use of 2D materials. A prominent representative is graphene, whose in-plane thermal conductivity is about 5300 W m^–1^ K^–1^, in contrast to its out-of-plane value which is lower than 10 W m^–1^ K^–1^.^27^ In three-dimensional materials, thermal anisotropies are comparably lower. High ratios of upper to lower thermal conductivity are reached for example by metallic wood^28^ (anisotropy ratio = 18) and layer-by-layer assembled nanofibrillated cellulose/graphene nanosheet hybrid films (in-plane thermal conductivity 12.6 W m^–1^ K^–1^, cross-plane 0.042 W m^–1^ K^–1^).^29^

A simple method to fabricate thermally anisotropic materials is the incorporation of unidirectionally aligned fibers in a polymer matrix.^30,31^ Such laminates show enhanced thermal conductivity in the fiber direction, whereas the heat transport orthogonal to the fibers is unfavorable.^32^ Carbon fiber composites typically develop anisotropy ratios of around 10. This is due to the high thermal conductivity of carbon fibers, which is orders of magnitude higher than that of the polymer matrix.^33^ Higher ratios are accessible by changing the fiber type to, e.g., metallic fibers, in which electronic transport is also possible.^34^

The central idea for controlling heat flow is to design thermally conductive paths.^35,36^ Anisotropic composites (or thermal metamaterials) are one method for creating such paths in heterogeneous media. Fibers with high thermal conductivity are well-suited for this task owing to their intrinsic anisotropy and ease of alignment in composite structures.^37^

This leads us to a novel concept for precise temperature distribution shaping using composite materials with thermally anisotropic building blocks. Carbon fiber laminates form the basis for the thermally anisotropic constituents. The heat flow within the individual constituents is controlled by their in-plane anisotropy. Their mutual arrangement and orientation will dictate the macroscopic heat transport. We demonstrate this concept via IR thermography and evaluate the key factors influencing the thermal transport in such anisotropic composites. In addition, we develop guidelines simplifying the design of composite structures to control heat transport. Similar to constructing a house out of toy bricks, we can fine-tune the thermal transport using the macroscopic thermal anisotropy of our individual laminates.

## Results and Discussion

### Thermal Anisotropy of the Laminates

The manufactured unidirectional (UD) aligned laminates with an overall porosity of 2.65% provide sufficiently high thermal anisotropy, which is verified by light-flash analysis (LFA). The highest thermal conductivity of (6.64 ± 0.33) W m^–1^ K^–1^ at 20 °C was obtained in the direction of the fibers. In contrast to this, the thermal conductivity orthogonal to the aligned fibers is with (0.40 ± 0.02) W m^–1^ K^–1^ comparably low. As a result, the anisotropy ratio of the laminates equals roughly 16. This anisotropy ratio is comparably high; recently fabricated materials exhibited anisotropy ratios ranging from 4 to 20.^24,28,38,39^ In some extreme cases of thin films or 2D materials like graphene, anisotropy ratios of 300 and larger could be achieved.^27,29^ However, these are of no use in the context of the study as these ratios are typically the result of high in-plane to through-plane thermal conductivity. In our case, in-plane anisotropy is the fundamental feature.

**Figure 1:**
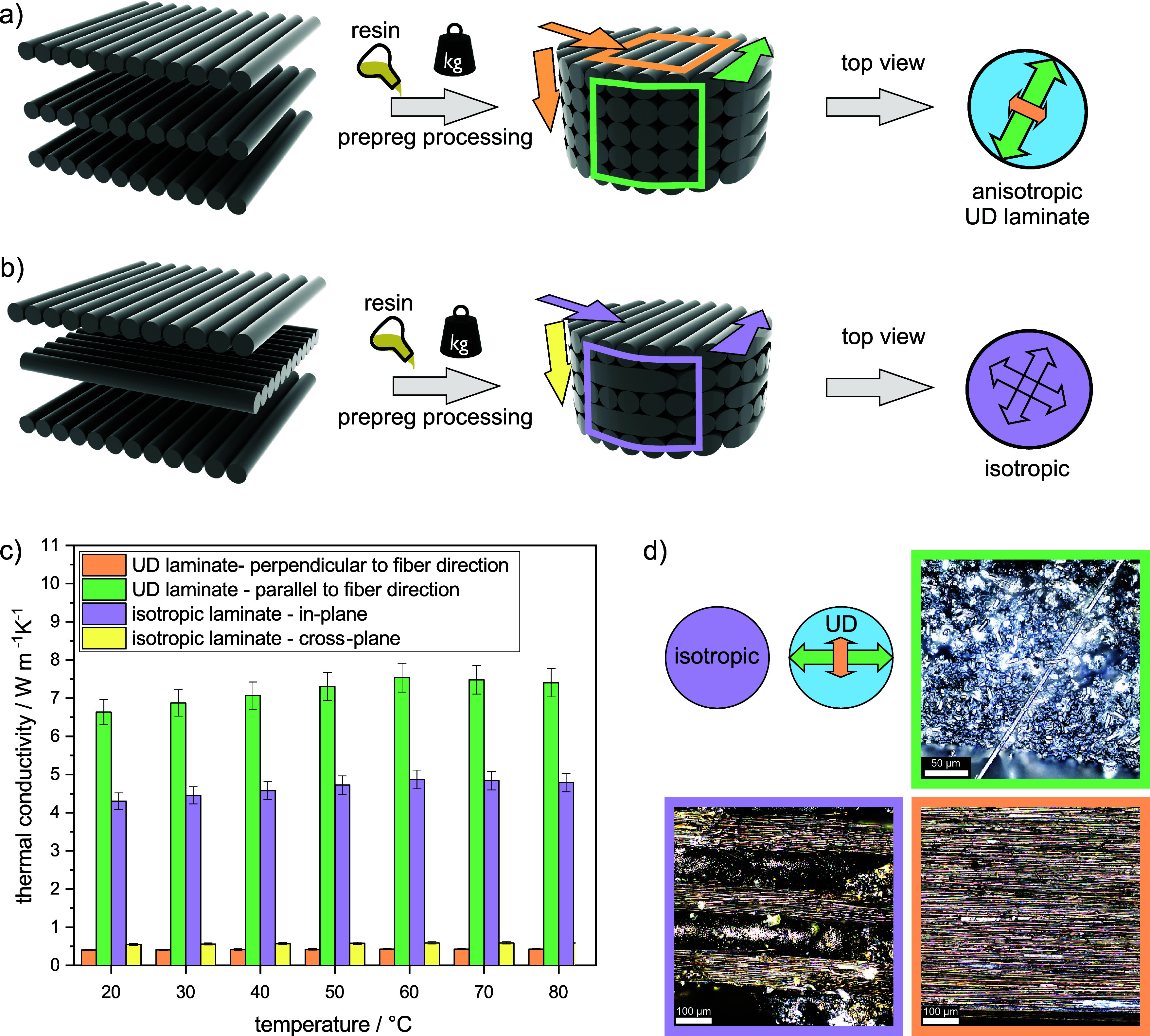
Schematical representation of the laminate manufacturing process. (a) Preparation of uniaxial aligned laminate with thermal anisotropy. (b) Preparation of biaxially stacked laminate with isotropic thermal conductivity. (c) Thermal conductivities of the laminates. The distinct orientations are highlighted by the respective arrows shown in (a) and (b). Fiber placing and stacking leads to a considerable reduction in thermal conductivity. (d) Laser scanning microscopy images of the respective laminates confirm the proposed alignment of the fibers within the fabricated laminates.

The thermal conductivity of the isotropic laminates made from biaxially stacked unidirectional prepregs is between the values of the anisotropic laminates. As expected it does not depend on the measurement direction within the thin disc plane. This confirms that the biaxial fiber arrangement outlevels the intrinsic thermal anisotropy of the fibers. A schematic of the fiber structures in the laminates is shown in Figure 1b, visualizing the biaxial stacking in the isotropic laminates. For the sake of clarity, they are referred to as isotropic in the remainder of this contribution.

Before designing complex composite structures, we examined the effect of anisotropy on the heat flow of single laminates. Therefore, discs with a diameter of 1 cm were cut from the laminate sheets. Then, the discs were subjected to a temperature gradient. In the case of the isotropic laminate, a homogeneous temperature profile between the hot and cold sides formed, which was independent of the rotation of the laminate disc. A slight curvature of the temperature isotherms can be observed in the vicinity of the disc’s edge. This is caused by the thermal boundary to air at the disc’s edge and the contact area between the disc and the heat sink and heat source. These form a ball cap rather than a contact point. Similar effects were also observed for the anisotropic discs.

**Figure 2:**
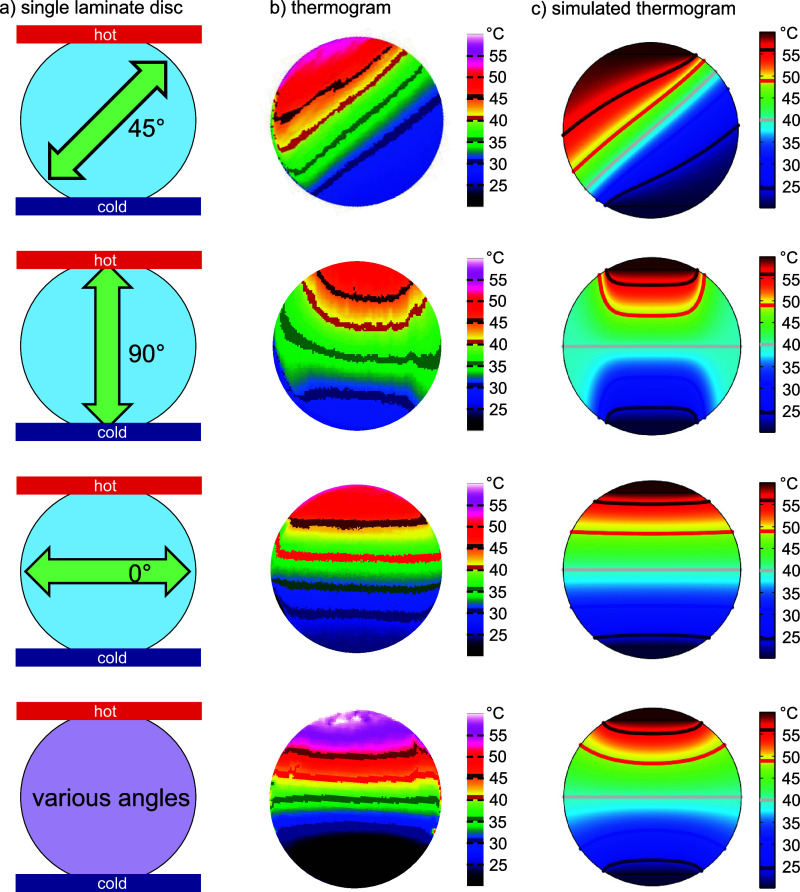
(a) Anisotropic (UD) laminate discs with different orientations of the fibers relative to the temperature gradient. The preferred direction of heat flow is denoted by an arrow. For comparison, the temperature distribution of isotropic discs is shown in the fourth row. It remains unaffected by the rotation of the disc. (b) Measured temperature distribution by IR thermography. (c) Calculated temperature distributions with COMSOL Multiphysics.

The temperature distribution of the thermally anisotropic UD laminates remarkably differs from the isotropic ones. Further, it strongly depends on the respective orientation of the fibers relative to the temperature gradient. If the axis of preferred thermal conduction is parallel to the temperature gradient, the heat is transported straight along the fibers. The heat transport to the edge of the discs is attenuated due to the presence of polymer resin between the individual fibers. In the case of perpendicular fiber orientation to the temperature gradient, the incoming heat is deflected vertically at each fiber. As a result, the temperature contour lines are oriented parallel to the temperature gradient. Other angles such as 45° deflect the heat in the respective orientation direction. The temperature profile reveals this deflection and thus the fiber orientation. Figure 2 shows the respective fiber orientation to the applied temperature gradient and the resulting temperature profiles. The respective steady-state temperature distribution measured with an IR camera and the corresponding FEM results match very well. A very good representation of the fiber orientation in a single laminate is given by isotherms (Figure 2b,c). They provide an excellent tool to compare thermograms from IR measurements with corresponding simulations.

We found that thermally anisotropic materials can influence the temperature distribution and, therefore, the heat flux inside the material. The controllable temperature distributions of the single laminates provide a profound basis for designing composite materials that can control the heat flux and the associated temperature distribution locally precisely.

### Design of Complex Laminate Structures To Control Heat Flow and Temperature Distribution

We demonstrate the capability to tailor the temperature distribution by distinctly oriented structures comprising several anisotropic and isotropic laminate discs. Figure S1 shows that the previously demonstrated effect on the temperature distribution is also valid in a composite structure. The bonding of the laminates with the thermal adhesive only slightly influences the temperature distribution. One droplet of thermal adhesive on each side of the structure gave reliable and reproducible results. Replacing more isotropic discs with UD platelets results in more distinct temperature gradients and makes it easier to control the temperature distribution of the composite material. UD platelets with the same orientation placed in one line combine their effects, channeling the heat more efficiently, compare also Figure S1.

**Figure 3:**
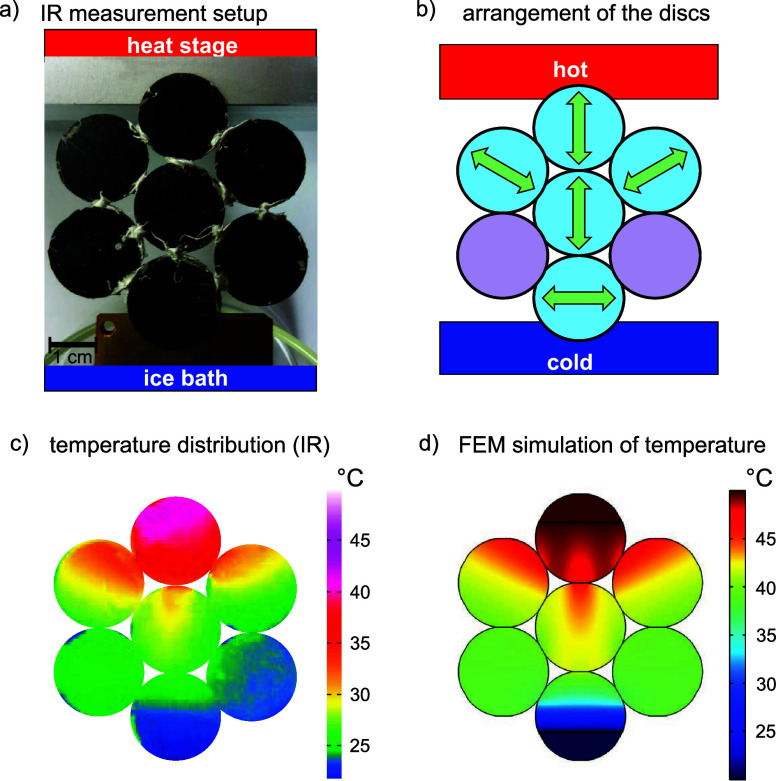
(a) Photograph of a laminate structure positioned between a heat source and heat sink for IR thermography. The arrangement and orientation of the single laminate discs are depicted in (b). (c) Measured temperature distribution with IR thermography. (d) FEM simulation of the temperature distribution for the laminate arrangement in (b).

One example of a more complex design is shown in Figure 3. Here, the heat is guided to the center of the structure, followed by an orthogonally oriented laminate disc. As a result, a sharp temperature gradient with more than 10 °C difference is created in this slice. We can also clearly distinguish between isotropic and anisotropic laminate slices. The anisotropic slices exhibit visible temperature gradients that indicate the orientation of the preferred conduction axis. The contact areas between the discs are relevant for the heat flow within such disc ensembles. The discs touch each other at distinct contact points. We facilitate the flow of heat across these contact points by the application of thermal glue, which provides a low thermal resistance and increases the actual contact area. Any source of thermal resistance, e.g., a small contact area or low-conducting glue, will alter the heat transport between the single constituents and, consequently, the observed temperature distribution. We attribute the slight asymmetry between the left and the right side as seen in Figure 3c to slight variations in thermal contact. A comparison to ensembles with maximized contact areas (hexagons or squares) is shown in Figure S5. In the case of the FEM simulations, the temperature distributions were derived from ensembles with well-defined contact areas. An unobstructed heat flow between the individual discs was assured by assuming no thermal resistance between the single discs. Thus, the temperature distribution in Figure 3 is fully symmetric. All in all, the experiment and the simulation agree very well, and we clearly show that thermal anisotropy significantly influences heat transport in composites.

Crucial for sharp temperature gradients (more than 10 °C difference) inside a structure are blockers. Blockers are UD platelets that are oriented orthogonal to the applied temperature gradient. Using blockers directly after a UD platelet oriented in the direction of the temperature gradient is very efficient in developing intense temperature differences inside the composite structure. The best results for controlling the temperature distribution are obtained when the UD laminates focus the heat from the hot side, leading to a single platelet in the middle of the composite structure. Some examples of the effect of single blockers are given in Figure S2. Placing blocker platelets underneath them creates a barrier for the heat flux—resulting in a sharp temperature gradient in the blocker platelet. One example is the temperature distribution in Figure 3c,d.

**Figure 4:**
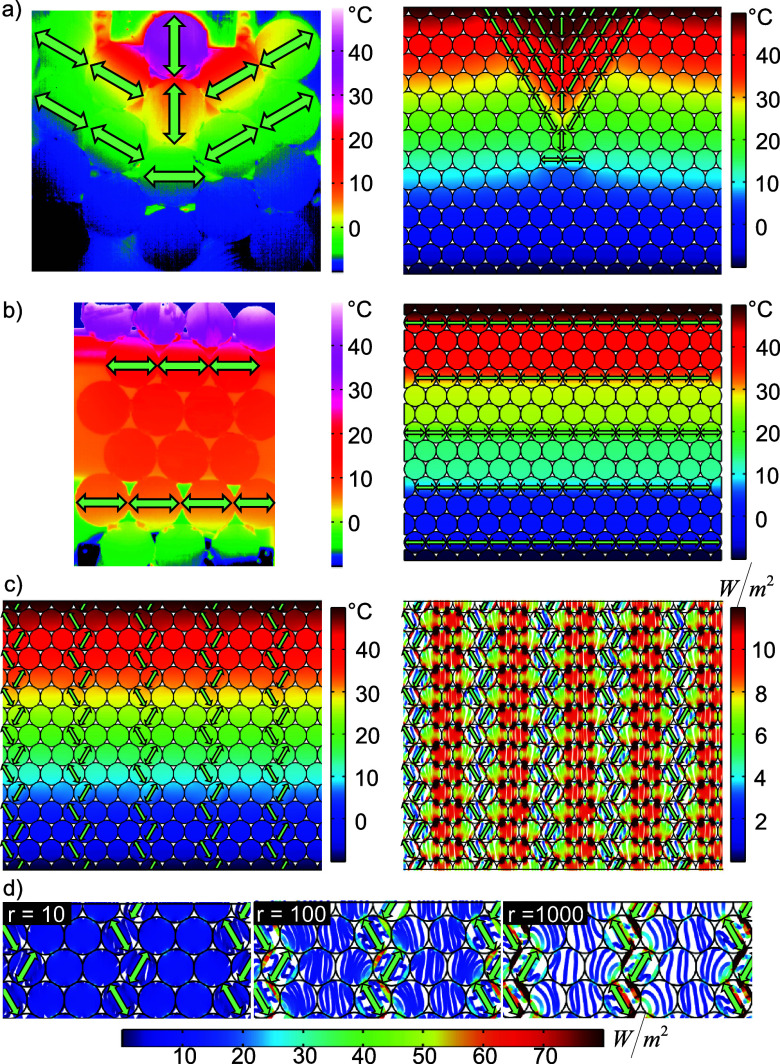
(a) IR thermogram (left) and FEM simulation (right) of a laminate structure designed to show a pyramidal temperature distribution. The green arrows mark the orientation of the anisotropic laminates. (b) Thermogram (left) and FEM simulation (right) showing the creation of isothermal zones. (c) FEM simulation of a material with heat channels created by the arrangement of the anisotropic discs, resulting in a homogeneous temperature distribution. On the right, the corresponding heat flux magnitude is depicted, revealing that the heat flows preferably through the isotropic discs. (d) Section of (c) for different ratios *r* of the thermal conductivity inside the anisotropic laminates. Note: all not-marked discs are thermally isotropic.

The use of blockers can be applied to macroscopic structures as well. We used FEM simulations to transfer the concept of tailored temperature distribution to macroscopic composite structures. Different large-scale composite structures are shown in Figure 4. We arranged them in a hexagonal lattice, as the previous seven-component structures also followed this lattice symmetry. The material in Figure 4a is designed to give a pyramidal shape to the temperature distribution. Therefore, the platelets are arranged in a way that guides all the incoming heat to the center of the structure, followed by a blocker line to ensure that sharp temperature gradients are maintained. The rest of the composite is composed of our isotropic laminate discs with moderate thermal conductivity.

A completely different design provides the arrangement in Figure 4b. Here, isothermal zones are created between horizontal lines consisting of UD platelets orthogonal to the temperature gradient. These blocker lines effectively block thermal transport in such a way that heat flow is reduced locally across the whole structure. After passing the blockers, thermal dissipation is fast until reaching the next blocker line. Thus, isothermal zones between the blockers are created, while the temperature differences required to achieve equilibrium under the given temperature gradient are concentrated on the blocker particles.

Both examples were experimentally realized using laminate discs. The fabricated laminate structures are limited in size due to the gluing process. Nonetheless, the IR measurements show good agreement with the simulations.

In contrast to the aforementioned structures, the large-scale composite structure in Figure 4c exhibits a temperature distribution that mirrors a continuous gradient from the hot to the cold side. Here, anisotropic platelets are incorporated into the structure without significantly impeding heat transfer. Hence, no distinct temperature distribution is visible.

Nevertheless, the heat flux magnitude reveals specific transport pathways inside the composite structure. The higher the absolute value of the heat flux magnitude, the more heat traverses a particular location. An analysis of the structure in Figure 4c reveals that the isotropic platelets channel the heat. The predominant heat flow occurs along the isotropic platelets straightforwardly through the structure. Two factors can explain this phenomenon. First, the anisotropy ratio of the laminate discs influences the channeling effect itself. The higher the anisotropy ratio, the more efficient the channeling will become. In our case, the anisotropy ratio of 16 is good but not superior. Second, the channeling in the presented structure increases the length of heat flow paths, counteracting the intended channeling effect. The thermal conductivity of the utilized isotropic laminates is of utmost importance for the current system. It is within the same order of magnitude as the higher thermal conductivity of the anisotropic laminates. Owing to the temperature gradient, the direction of the heat flow is predetermined. Faced with the choice between the nearly equally effective isotropic laminates and the anisotropic laminates guiding the heat through a longer transport path, the shorter route prevails.

If the isotropic components are replaced with a material possessing a significantly lower thermal conductivity, the anisotropic channel becomes the energetically favored pathway for heat transport. Tuning the properties of the isotropic constituents regarding the anisotropic ones enables us to have precise control over heat channeling. Likewise, the quantity of heat flowing through the channels can be fine-tuned. Figure 4d illustrates the impact of the anisotropy ratio in the anisotropic discs on thermal transport within the structure. As the anisotropy ratio (high- vs low-conducting) increases from left to right, only the thermal conductivity along the preferred conduction axis (along the fibers) is enhanced. At *r* = 10, the thermal conductivity of the isotropic laminates is comparable to that of the preferred axis in the laminates. Elevating the thermal conductivity of the conductive part (along the fibers) results in changes in the heat flow. At *r* = 100, the heat flow along the channels becomes favorable and at *r* = 1000, the major part of thermal transport occurs along the defined channels. A similar effect is obtained when decreasing the thermal conductivity of the isotropic particles surrounding the heat channels.

This simple experiment shows that thermal transport within thermally anisotropic composites is influenced by (1) the intrinsic ratio of anisotropy of a material and (2) the thermal properties of the surrounding matrix (if present). With these key parameters, advanced structures to control heat transport can be designed. The orientation of the anisotropy, in combination with the arrangement, makes it possible to generate a variety of tailored structures, either imposing a certain temperature distribution or manipulating the heat flow.

In addition, the shape of the anisotropic building blocks provides an additional degree of freedom in creating targeted thermal transport. The shape of the building block determines how the heat flows through the material. In squares or rectangles, the applied temperature conditions reach across the whole structure. Under adiabatic conditions at the boundaries parallel to the temperature gradient, this leads to heat accumulation at certain angles of the preferred conduction axis (Figure S4). Contrary to that, this does not happen in our discs or hexagons. Here, at the right and left additional material exists, allowing for more tortuous thermal paths. An even more intriguing effect is due to the arrangements or packing of the building blocks. Spherically shaped constituents (discs, spheres, ellipsoids, etc.) form lattices or packings with holes inside. If the components matrix is air or polymer-based, nonconducting holes form, preventing heat transport across the holes. The size and shape of the holes can be adjusted by the contact area and packing structure. In addition, the limited contact areas between the spherical building blocks limit the options of short thermal paths through the structure. In the case of blocks or squares as well as hexagons (in two dimensions), lattices without holes can be formed. An overview of different 2D temperature distributions for various shapes and arrangements of building blocks is provided in Figure S5. Naturally, they differ from the previously shown temperature distributions in a hexagonal lattice. In all cases, it is possible to manipulate temperature distributions and heat flow in the composite structure. Every shape provides its specific temperature distribution when subjected to a temperature gradient, leading to differences in the thermal transport within the composite.

Combining the different specifics of building block types, a whole library for targeted thermal transport opens up. The concept can be extended to three-dimensional objects as well. Here, attention to materials that show thermal anisotropy in more than two directions may open up even further possibilities. Again, the thermal conductivity of the matrix can be used as an additional tuning parameter.

### Internal Heat Sources

**Figure 5:**
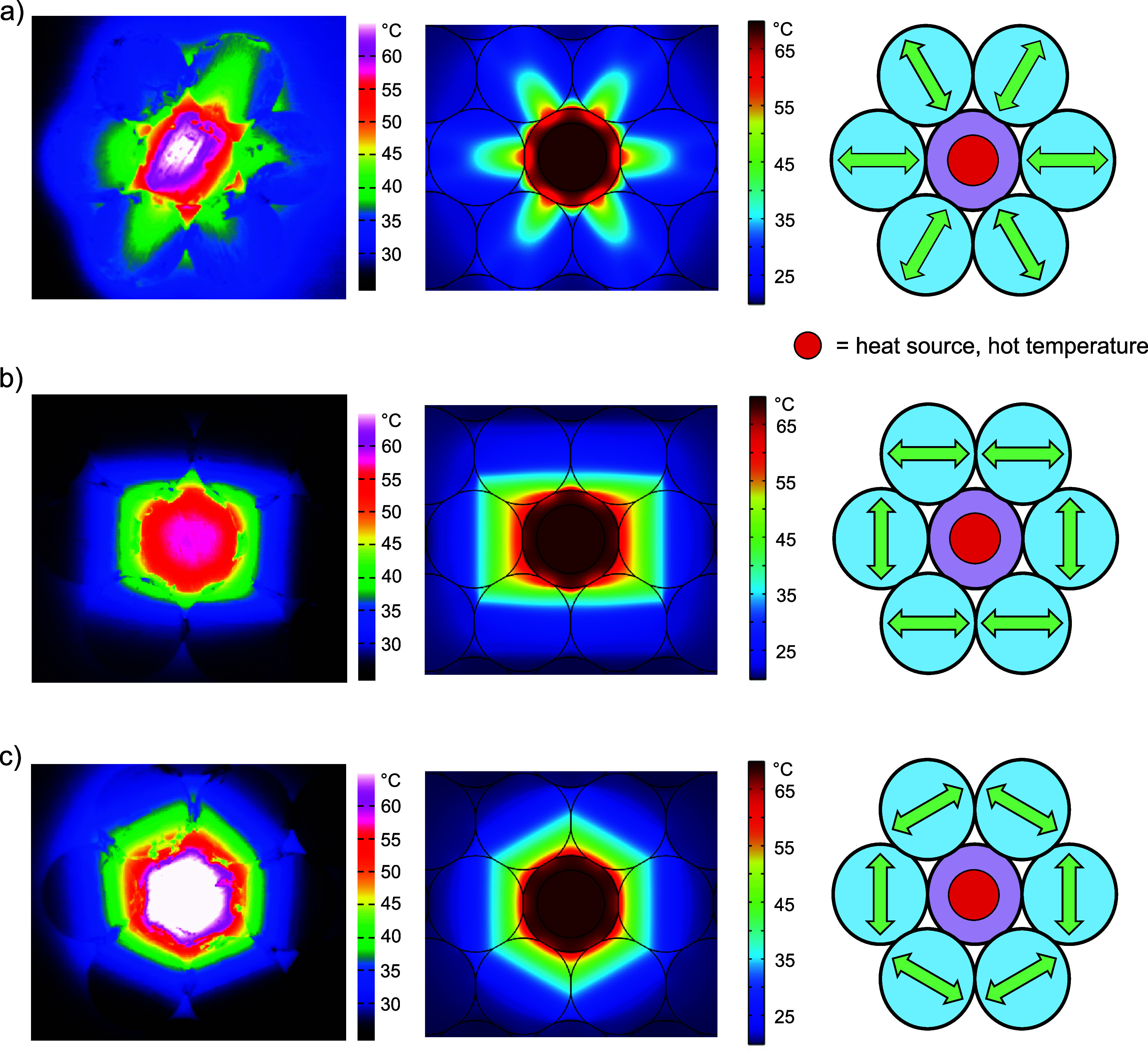
(a) Thermogram and FEM simulation of an anisotropic composite structure designed to dissipate heat from the internal heat side to the outer edges by the UD laminates. The corresponding arrangement of anisotropic laminates is given on the right. (b) Rectangular heat cage, validated by IR measurement on the left and the calculated temperature distribution in the middle. (c) Thermogram and FEM simulation of a heat trap with a hexagonal-shaped temperature distribution.

In many applications, internal heat sources are embedded in a composite structure. Consequently, we replaced the applied temperature gradient with an internal heat source implemented in a thermally anisotropic composite material. We demonstrate three scenarios for designing an anisotropic environment around a localized heat source. The first example in Figure 5a is designed to dissipate the heat as efficiently as possible via heat channels. Arranging the channels to lead the heat away from the heat source leads to a lower temperature at the center disc. We adjusted our experimental setup to monitor the temperature distribution using IR thermography. The center of the laminate structure is heated by a copper cylinder connected to the heat stage. As expected, the measurements confirm the FEM simulation results, where the temperature spreads radially away from the heat source. Only the top-left particle represents an exception, which we attribute to a compromised thermal contact with the core.

Conversely, the heat can be confined in anisotropic cages, using our constituents’ thermal anisotropy. Figure 5b,c illustrates two distinct hexagonal arrangements of laminate discs surrounding an isotropic platelet. The structure’s center is consistently maintained at 50 °C. In the experimental setting, thermal equilibrium was achieved after a few minutes, and the temperature distributions shown in Figure 5 remained unaltered, regardless of the heating duration. This experiment demonstrates that a heat trap can be realized with constituents possessing a moderate anisotropy, such as the laminates employed here.

The heat dissipation in both structures is influenced by the orientation of the anisotropic platelets. The first structure is designed to dissipate heat from the center to the left and right boundaries of the structure. As a result, the temperature in the middle disc is lower than the one subjected to the heat traps in Figure 5c. Note that the experimentally measured temperatures are sensitive to the tilting of the single platelets and the contact between the heat source as well as between the platelets. Therefore, absolute temperature comparisons will only be undertaken from the FEM data.

Further, subtle differences arise due to the cage shape. The hexagonal cage blocks the heat flow more efficiently than the rectangular cage. As a consequence, the temperature at the center is slightly enhanced (4 °C) in comparison to the rectangular cage. The hexagonal arrangement of the platelets supports the hexagonal cage. If squares replace the discs, the rectangular cage is the optimal choice for trapping heat around a single building block.

We want to end our discussion with a few notions on the relevance of anisotropic heat conductors and potential areas of use. The role of anisotropy has been appreciated in thermal metamaterials, and particularly concepts for thermal cloaking. Inverse to the heat cages shown above, in these cases the flow of heat into distinct areas can be suppressed by sufficiently anisotropic environments.^40−42^ Nevertheless, thermally anisotropic materials offer a much wider variety to shape and control heat flow as we demonstrated here. For example, heat dissipation is of uttermost importance to ensure the safe operation of batteries and electronic devices^43,44^ and is also relevant for LED cooling.^45^ In these cases, anisotropy offers great possibilities to dissipate heat via well-conducting materials. It also allows for the protection of sensitive parts from excessive heat exposure. Yet, the concomitant evolution of temperature gradients needs to be finely balanced,^46^ which may induce unwanted stress on the device of interest.

In an extreme case, uneven heat flow distributions can lead to the formation of hot spots, which is critical for most electronic applications. Up to date, the cooling of electronics is addressed by the design of micro/mini-channel heat sinks.^47^ Common strategies address the optimization of different types and material combinations for micro channeling.^44,48−50^ Here, the intentional use of thermal anisotropic materials presents another possibility to channel the waste heat to efficient heat sinks.

## Conclusion and Outlook

We studied the intricacies of temperature distributions in composite structures, exploring the influence of anisotropic elements and innovative designs for heat management. For this purpose, we used laminate discs possessing a distinct thermal anisotropy. Our chosen material was a carbon-fiber laminate, facilitating the fabrication of both thermally anisotropic and isotropic laminates. The heat flow within individual constituents is influenced by the in-plane anisotropy, significantly affecting the temperature distribution when subjected to a temperature gradient.

The orientation of the fiber axis at different angles to the temperature gradient had a profound influence on the heat transport in the anisotropic disks. Leveraging this effect, we developed macroscopic composite structures to locally direct the heat flow. We applied IR thermography to reveal the temperature distributions of the various composite structures. Finite element method (FEM) simulations provided support, demonstrating that heat flux control could be extended to larger composites and micron-size scales.

We identified three key factors for fine-tuning thermal transport in the composites: (1) the thermal anisotropy ratio within the building blocks determines the extent to which thermal transport in the constituent is affected. Surprisingly, materials with anisotropy ratios greater than ten are sufficient to achieve noticeable effects. Anyhow, higher anisotropy ratios result in more pronounced control of the heat flow in the composites. (2) The orientation of the anisotropy axes of the building blocks relative to the temperature gradient. The temperature distribution and, therefore, the heat flow in an anisotropic material depends strongly on its orientation. Thus, the anisotropic building blocks can be strategically used to enhance heat dissipation or block heat flow. (3) Further, isotropic materials significantly influence thermal transport in composites. Their thermal conductivity plays a crucial role in determining whether thermal transport occurs through the anisotropic constituents. Consequently, a low-conductivity matrix of isotropic building blocks allows for more precise control of heat flow.

To sum up, we successfully developed a method to control thermal transport locally in composite structures. The concept allows for fine-tuning thermal transport using the macroscopic thermal anisotropy of individual laminates. Transferring the idea to nano- or microstructuring such as 3D printing applications makes it possible to control thermal transport at the micro- as well as the macrolevel.

## Experimental Section

### Materials and Methods

#### Fabrication of Laminates

The unidirectional (UD) prepregs were produced via a hot melt processing route at the prepreg machinery of the University of Bayreuth. The resin system is based on DGEBA epoxy resin with Dicyandiamide as a curing agent and urea accelerator. IMS-65 24K carbon fiber rovings from Teijin Carbon Europe GmbH (Wuppertal, Germany) with an areal weight of 140–150 gsm were used for prepreg manufacturing. The laminate was hand-laid with 9 layers to achieve 1 mm thickness. After stacking it was cured under autoclave conditions at 6 bar at 80 °C for 1 h and 135 °C for 4 h. The laminate sheets were cut into discs with the Diadrive 2000 CNC milling machine from Mutronic Präzisionsgerätebau GmbH & Co.KG (Rieden am Forggensee, Germany).

#### Characterization of Laminates

Thermogravimetric measurements were conducted with a TG 209 F1 Libra (Netzsch-Gerätebau GmbH, Selb, Germany) to determine the fiber volume content based on the standard DIN16459. Therefore, the samples of the laminate were first dried for 2 h in the TGA at 120 °C under an air atmosphere and then heated up to 450 °C with a heating ramp of 10 K min^–1^ under a nitrogen atmosphere. Finally, 450 °C was kept for 170 min. The fiber volume content was determined by the remaining mass of the laminate in comparison to neat fibers after the cycle to 61 vol % ± 1.

Laser scanning microscopy was performed with a LEXT OLS5000-SAF microscope (Olympus) to analyze the fiber structure in the laminate samples. The measurement directions with regard to the sample are indicated in Figure 1.

The thermal diffusivities of the laminates were analyzed by light flash analysis (LFA) (LFA 467 HT HyperFlash, Netzsch). The through-plane thermal diffusivity of the anisotropic laminates was determined with the standard sample holder via LFA. We reasonably assume that the through-plane thermal diffusivity equals the thermal diffusivity perpendicular to the oriented carbon fibers within the disc plane. The in-plane thermal diffusivity parallel to the fiber direction was also measured by LFA. Therefore, the laminates were cut, turned 90° and glued together. After polishing the top and bottom sides, the standard holder of LFA was used to determine the thermal diffusivity. Similarly, the in-plane thermal diffusivity of the isotropic laminates was determined. The density was determined by a helium pycnometer (Quantachrome Ultrapyc 1200e). The heat capacity was determined by differential scanning calorimetry (Discovery 2500, TA Instruments). The thermal conductivities are obtained by multiplying the density, heat capacity, and thermal diffusivity.

#### Manufacturing of the Composite Laminate Structures

The laminate discs are glued together with a two-component thermal glue (Quick Cure Silver Epoxy QC-WLK-CQ-07 Part A, Arctic Silver Inc., Quick-Ohm Küpper & Co. GmbH) in the predestined arrangement and orientation. First, the contact surfaces of the two neighboring discs were coated with adhesive. Then the panels were pressed together. After a short drying time, another drop of adhesive was applied to the contact surface from both sides (top and bottom) of the construction. This ensured that the composite discs were bonded together with a sufficient amount of adhesive and that a sufficiently conducting contact area formed between adjacent discs. Finally, the laminate composite structures were dried at room temperature for 24 h.

#### In Operando Measurement of the Temperature Distribution in the Composite Structures

To measure the temperature distribution in the laminate structures, a home-built setup consisting of a heating stage (Präzitherm, Harry Gestigkeit GmbH Düsseldorf), an ice bath, and the IR camera (VarioCAM HD 4300 by Infratec) was used. The sample was placed at the edge of the heating table, which was kept at 50 °C. The opposite edge of the structure was placed on a copper block cooled by an ice bath to a temperature of about 2 °C. The thermal contact between the laminate structure and the respective hot and cold sides was improved with heat-conducting paste (RS Heat Sink Compound Plus, RS Pro). Thus, a quasi-stationary temperature gradient across the free-standing sample was generated. The temperature distribution of the sample was measured after 30 min ensuring a steady-state temperature distribution. A photograph and schematic of the setup is provided in Figure 3.

#### Finite Element Method

Using the heat transfer module of COMSOL Multiphysics (Version 6.1), the stationary heat transport in the laminate structures was modeled. Therefore, two-dimensional models of the disc-based structures were made. The experimentally obtained thermal properties of the laminates serve as input parameters for the simulations of the disc arrangements. Details of the implementation of the anisotropic thermal conductivity are given in ref (46). It was assumed that the discs were slightly pressed together. Hence, the contact line between the discs was modeled as a straight line between two adjacent discs. This contact line amounted to about 5% of the circumference of the disc. No thermal resistance was assumed between the two discs. This results in a smooth mesh throughout the structures, see Figure S3. The laminate structures were subjected to a temperature gradient, defined by constant temperature sources. In Figure 2, we used 293,15 K for the lower temperature and 333,15 K for the hot side. In Figure 3, the upper temperature was adjusted to 323,15 K, while 293,15 K was chosen for the lower bound. For the structure shown in Figure 4, we used 263,15 K and 323,15 K, respectively. In Figure 5, the outer boundaries were set to 293,15 K. In addition, the heat source in the middle of the structures was represented by a circular area set to a constant temperature of 343 K.

## Supporting Information Available

Detailed explanation of the heat transport for various arrangements, with a focus on the implications of blockers on thermal transport, and simulation data of nondisc-shaped building blocks (PDF)
