# Supplementary material for: Shaping Thermal Transport and Temperature Distribution via Anisotropic Carbon Fiber Reinforced Composites
Source: ACS Omega. 2024 Sep 4;9(37):39232–41. doi: 10.1021/acsomega.4c06558 (PMC11411524; doi:10.1021/acsomega.4c06558)
Supplement: Supplementary file 1 — ao4c06558_si_001.pdf [file ao4c06558_si_001.pdf]

Supporting Information
Shaping Thermal Transport and Temperature Distribution via Anisotropic Carbon Fiber Reinforced Composites

Flora Lebeda,[†,‡] Martin Demleitner,[¶] Annalena Pongratz,[¶] Holger Ruckdäschel,[¶,§] and Markus Retsch[*,†,‡,‖]

†*Department of Chemistry, Physical Chemistry I, University of Bayreuth, Universitätsstraße 30, 95447 Bayreuth, Germany*

‡*Bavarian Center for Battery Technology (BayBatt), Weiherstraße 26, 95448 Bayreuth, Germany*

¶*Department of Polymer Engineering, University of Bayreuth, Universitätsstraße 30, 95447 Bayreuth, Germany*

§*Bavarian Polymer Institute, Bayreuth Center for Colloids and Interfaces, and Bayreuth Institute of Macromolecular Research, Universitätsstraße 30, 95447 Bayreuth, Germany*

‖*Bavarian Polymer Institute, Bayreuth Center for Colloids and Interfaces, Universitätsstraße 30, 95447 Bayreuth, Germany*

E-mail: markus.retsch@uni-bayreuth.de

Phone: +49 921 55 3920

Supplementary Information

Shape and arrangement of building blocks

Instead of discs, one may use blocks, hexagons, or other shapes suited for specific needs. Figure S5 gives an overview of several composite structures for three types of building blocks. Discs offer the most variability in their arrangement, they can either be distributed in a simple cubic lattice or a hexagonal lattice. Additionally, randomly packed structures are most easily achieved using discs (or spheres in 3D, respectively). Starting with a simple cubic arrangement of discs, one easily obtains a square lattice made of squares by increasing the sphere radius until they overlap completely. In the same way, the hexagons can be created on the base of discs arranged in a hexagonal lattice. The temperature distributions, however, distinguish a lot from each other. In the isotropic case (top row of Figure 5, the influence of non-conducting pores in a material is visible. For the squares and hexagons, the temperature distribution is nearly perfectly homogeneously distributed between the hot and cold edges. In the case of the discs in a cubic simple arrangement, the heat has to flow through the narrow contacts between the particles, resulting in a slight change in the temperature distribution around the contact points. The heat transport to the sides is in all cases negligible, as the temperature gradient imposes the direction of heat flow from top to bottom. A similar effect is found in the disc structure in a hexagonal lattice. The introduction of a parallel line of anisotropic building blocks leads to minor effects on the respective temperature distributions. Conversely, the implementation of only two anisotropic building blocks in the left corner (orientation 45° influences the temperature distributions drastically. As expected, for the squares and hexagons the temperature distributions display the orientation of the anisotropy directly. In the case of the discs arranged in a hexagonal lattice, the anisotropy of the middle disc is also directly visible. The temperature distribution strongly resembles that of the hexagons, with small additional effects imposed by the non-conducting spaces between the discs. Similarly, the temperature distribution of the discs arranged in a square lattice resembles the one of the squares. However, the amount and orientation of

anisotropic discs can not be seen at a direct glance. Similar observations are found in the case of the structures with purely anisotropic components.

To sum up, the shape and arrangement of the building blocks can be chosen freely. Each of them results in a slightly different temperature distribution that is mainly determined by the position and broadness of the contact areas between the single building blocks.

Heat flow in a single square: angle dependency of anisotropy

We observed a very interesting artifact when analyzing the different types of building blocks in detail at the component level. Beginning with a simple square with thermal anisotropy, the following temperature distributions are obtained, depending on the respective orientation of the preferred conduction axis (see Figure 4. Note that the temperature gradient here is imposed from right to left, for reasons of simplicity regarding the notation of the angles. The results for 0° as well as for 90° are homogeneous temperature distributions, as expected. However, for angles between 0° and 45° unexpected artifacts occur. Naturally, the heat is conducted along the axis of preferred heat conduction, but as long as the angles are such that the heat is led to the thermally insulated sides of the square, the main heat flow occurs on the left side, being the shortest path using the unfavorable bad conductivity axis. A detailed look at the boundaries of the temperature gradient reveals that there are regions close nearby with enhanced temperature. We found the highest, and respectively lowest temperature region for 45°. Here, both maxima exceeded the applied temperature conditions for the formation of the steady-state temperature gradient. The same effect can be found for rectangles, however at different angles, depending on the aspect ratio of the rectangle. For angles larger than the diagonal through the block, the heat transport can follow unhindered the direction led by the preferred conduction axis. Consequently, the effect of heat accumulation is not observed anymore.

Presumably, this effect only shows up for completely thermally isolated systems with a shape that blocks the heat transfer from top to bottom completely. We did not observe heat

accumulation for discs, ellipsoids, and triangles under the same conditions. Under normal circumstances, convection and radiation will take place. As a result, the effect will vanish. Nonetheless, the temperature distribution inside the building block will be guided by internal anisotropy.

Supplementary Figures

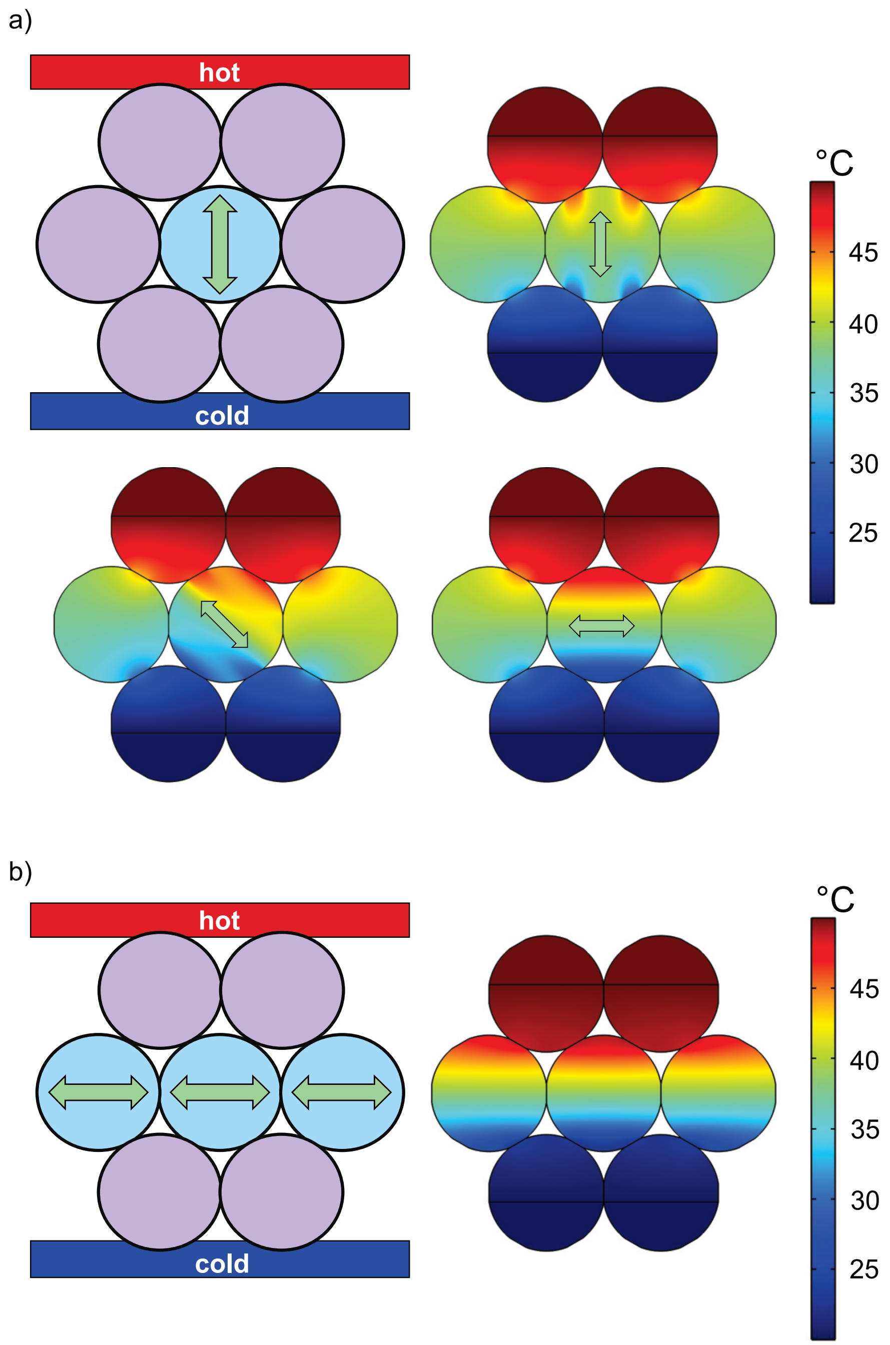

Figure S1: a)Thermograms of single anisotropic discs surrounded by six isotropic laminate discs. The temperature distribution is changed locally by the orientation of the preferred conduction axis of the anisotropic disc.b) Temperature distribution of a line of neighbored discs with anisotropy orthogonal to the temperature gradient.

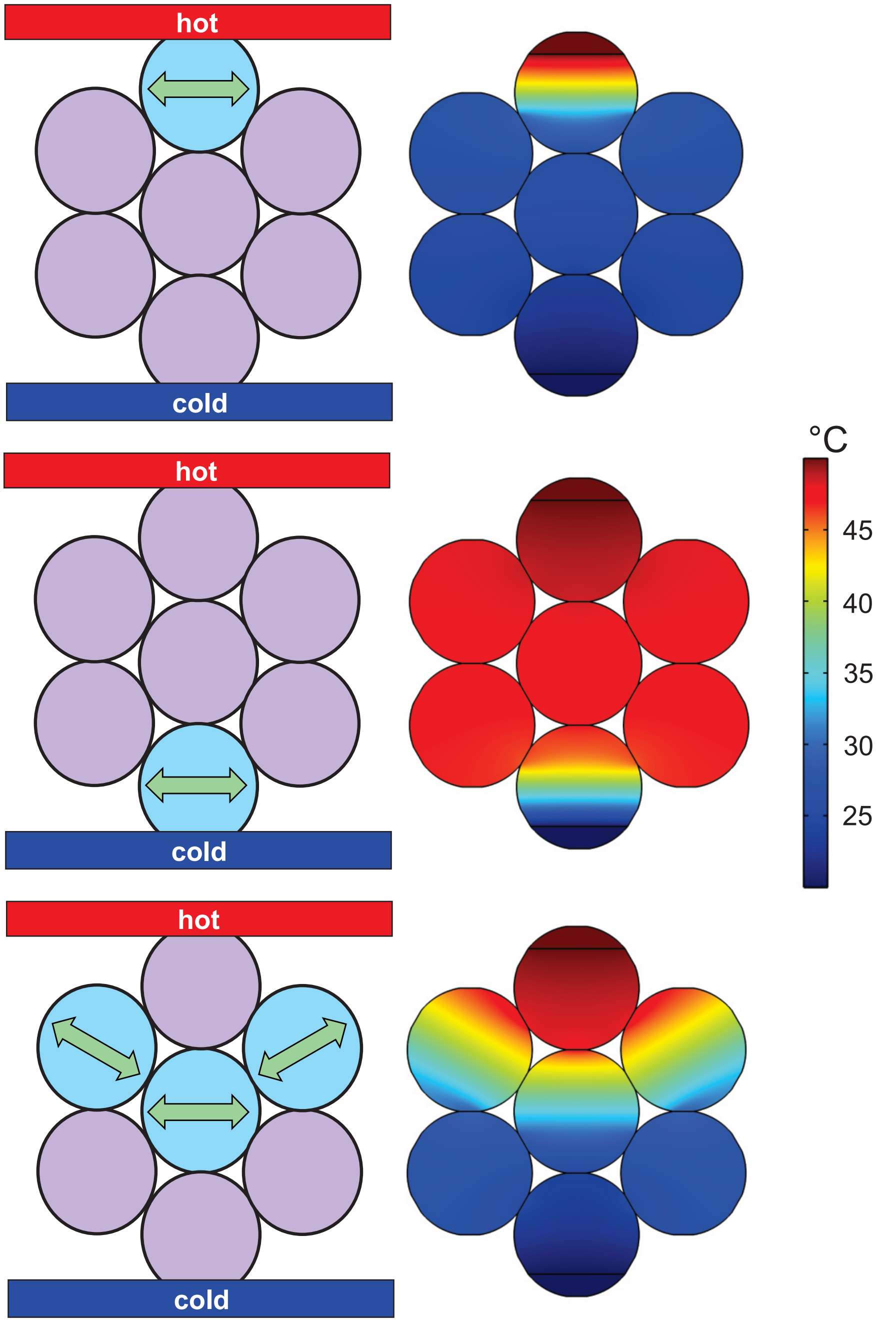

Figure S2: Thermograms illustrating the strong effect on the temperature distribution of blockers (discs with the preferred thermal conduction orthogonal to the temperature gradient. The use of blockers leads to strong local temperature gradients in single building blocks.

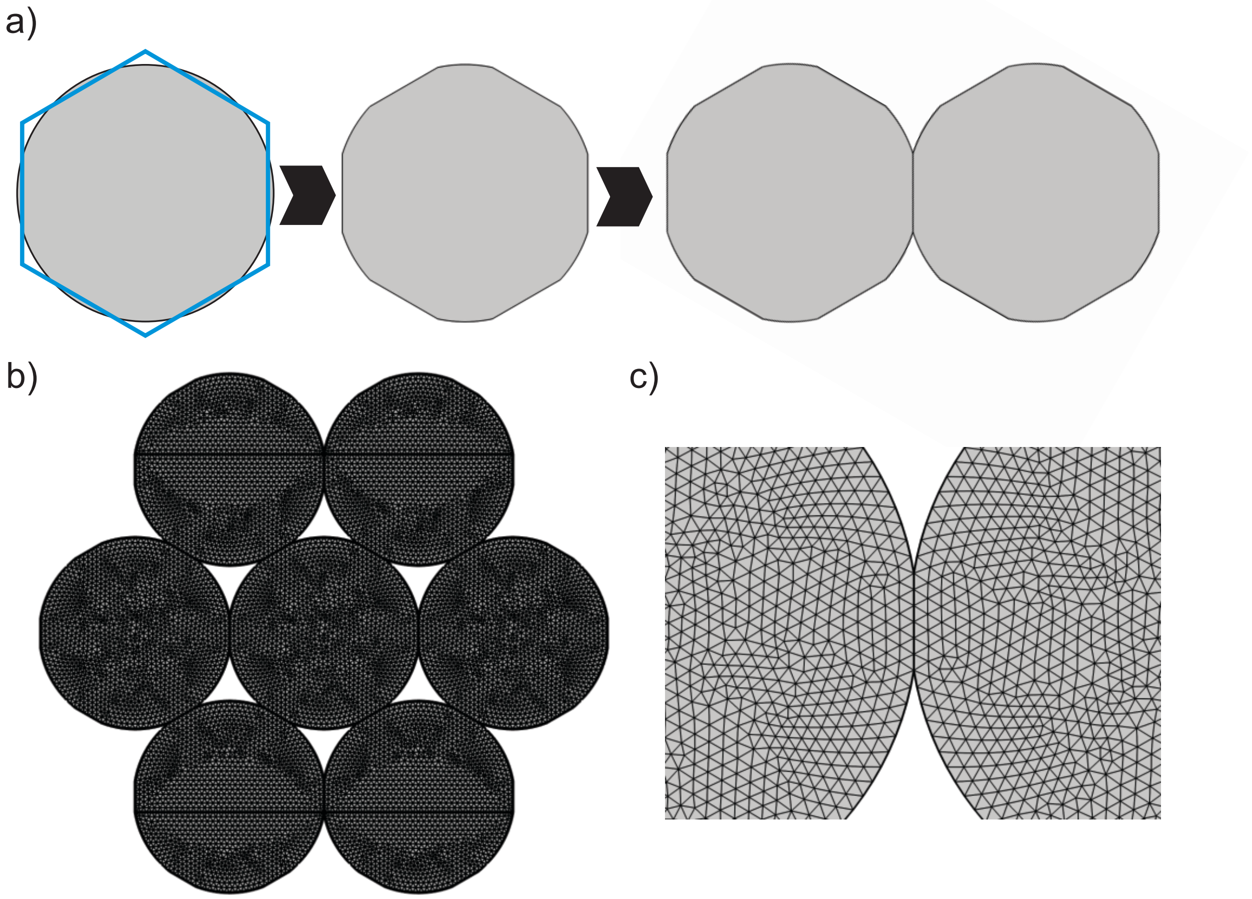

Figure S3: Construction of the contact areas between the discs for the FEM simulations. a) Spherical disc combined with a hexagon. After subtraction of the hexagon, a disc with six flattened sides is obtained. Placing a second disc next to it results in a flat contact area between the discs. In reality, this corresponds to particles that are pressed together. In the experiments, thermal adhesive is used to contact the discs, introducing an interface between the respective discs. b) shows the FEM mesh of a 7-compound structure, whereas c) shows the mesh at a disc contact in more detail. Note that only b and c show the employed contact line in the correct size ratio (5 % of the circumference). For a), the contact was expanded to enhance visual understanding of the creation process.

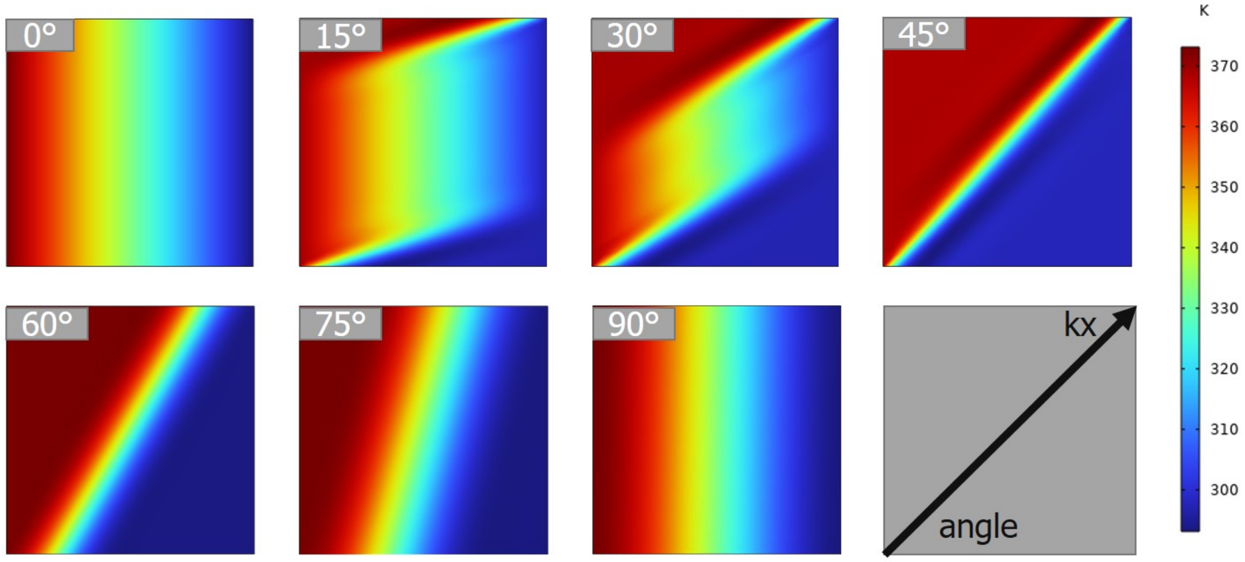

Figure S4: Temperature distributions of a single anisotropic square for different angles of the preferred thermal conduction axis.

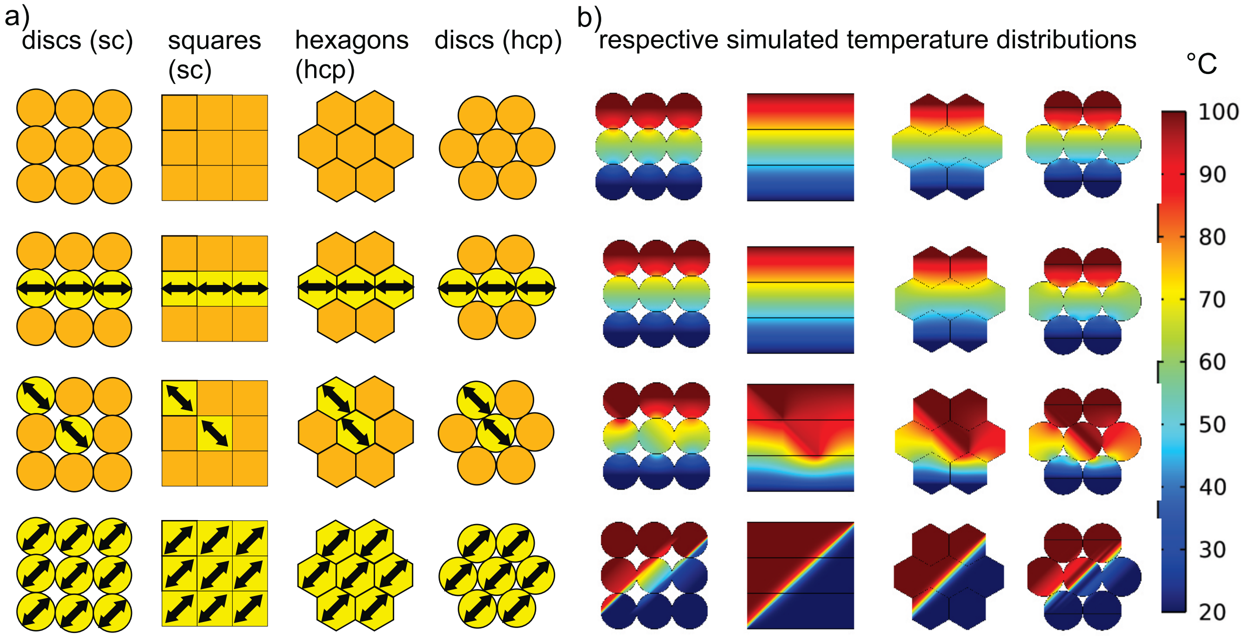

Figure S5: A variety of building blocks in different arrangements are shown in a). The corresponding temperature distributions are depicted in b). The top row shows completely isotropic arrangements, whereas, in the bottom row, all building blocks possess internal thermal anisotropy, with an orientation of the preferred conduction axis of 45°.
